# Supplementary material for: Chromosome-level haplotype-resolved assembly of highly heterozygous grass genomes with PhaseGrass
Source: Nat Commun. 2025 Dec 20;17:12. doi: 10.1038/s41467-025-66377-5 (PMC12764793; doi:10.1038/s41467-025-66377-5)
Supplement: Supplementary file 1 — Supplementary Information [file 41467_2025_66377_MOESM1_ESM.docx]

Supplementary Fig. 1. Estimated genome size, uniqueness and heterozygosity generated with GenomeScope2 for a, the *Lolium perenne* gentopye DH647 and b, the *L.* *multiflorum* genotype Sikem based on 21-mers from whole genome sequencing short reads. In each plot, the value of “len” indicates the estimated genome size, the value of “uniq” indicates the proportion of nonrepetitive sequences and the value of “ab” indicates the level of heterozygosity. Source data are provided as a Source Data file.

Supplementary Fig. 2. Integrated Genome Browser (IGV) screenshot of DH647 phasing results. a, Small phase blocks of DH647 on Kyuss pseudo-chromosome (Chr) 5. The chromosome-level phase block was indicated by the black arrow, and the small phase blocks were indicated by red arrows. No Hi-C phased SNPs were observed in the small phase blocks, and no ONT reads covering these small blocks were long enough to bridge the upstream and downstream SNPs in the chromosome-level phase block. Therefore, these small phase blocks could not be integrated into the chromosome-level phase block. b, Alignment-based read binning may not assign long reads to a haplotype due to reference bias. ONT reads were assigned to different haplotypes (indicated by red and blue colors in the ONT alignment track) by WhatsHap. Reads colored gray could not be assigned to a haplotype. The unassigned reads indicated by the red arrow might be mistakenly mapped to this repetitive region (reference bias) as the read alignment coverage was much higher compared to the neighboring regions. These unassigned reads did not span more than 1 SNP, thus they were not assigned to a haplotype by WhatsHap. However, if these reads indeed contain some haplotype-specific k-mers, then haplotype of these reads could be determined with the k-mer-based binning method.

Supplementary Fig. 3. K-mer profile of DH647, Sikem and HEN17. a-c, K-mer profile of DH647 hapolme 1, haplome 2 and diploid assembly, respectively. d-f, K-mer profile of Sikem hapolme 1, haplome 2 and diploid assembly, respectively. g-i, K-mer profile of HEN17 hapolme 1, haplome 2 and diploid assembly, respectively. The k-mer profile plot shows the comparison between k-mers from WGS short reads and k-mers from the assembly, and the aim of this comparison is to check if the assembly is correct or complete. For a heterozygous diploid genome, two major peaks should be observed with the first peak indicating k-mers from heterozygous regions (referred to as het-mers hereafter) and the second peak indicating k-mers from homozygous regions (referred to as hom-mers hereafter). The x-axis indicates the coverage of the unique k-mers in the reads, and the coverage of her-mers should be the half of the hom-mers. Colors in the peak indicate the copy number of a k-mer in the assembly. Het-mers in the assembly should have 1 copy (red color in the plot), hom-mers in the assembly should have 2 copies (purple color in the plot). Black color suggests that a k-mer is present in the reads but absent in the assembly. For a correct and complete haploid assembly, the first peak should be covered by half red and half black, and the second peak should be covered all by red. For a correct and complete diploid assembly, the first peak should be covered all by red, and the second peak should be covered all by purple. Red color in the second peak indicates that some hom-mers only have one copy in the diploid assembly. The k-mer profile of DH647 diploid assembly showed more red areas in the second peak. This is very likely because the less heterozygous regions on chromosomes 1 and 4 were collapsed.


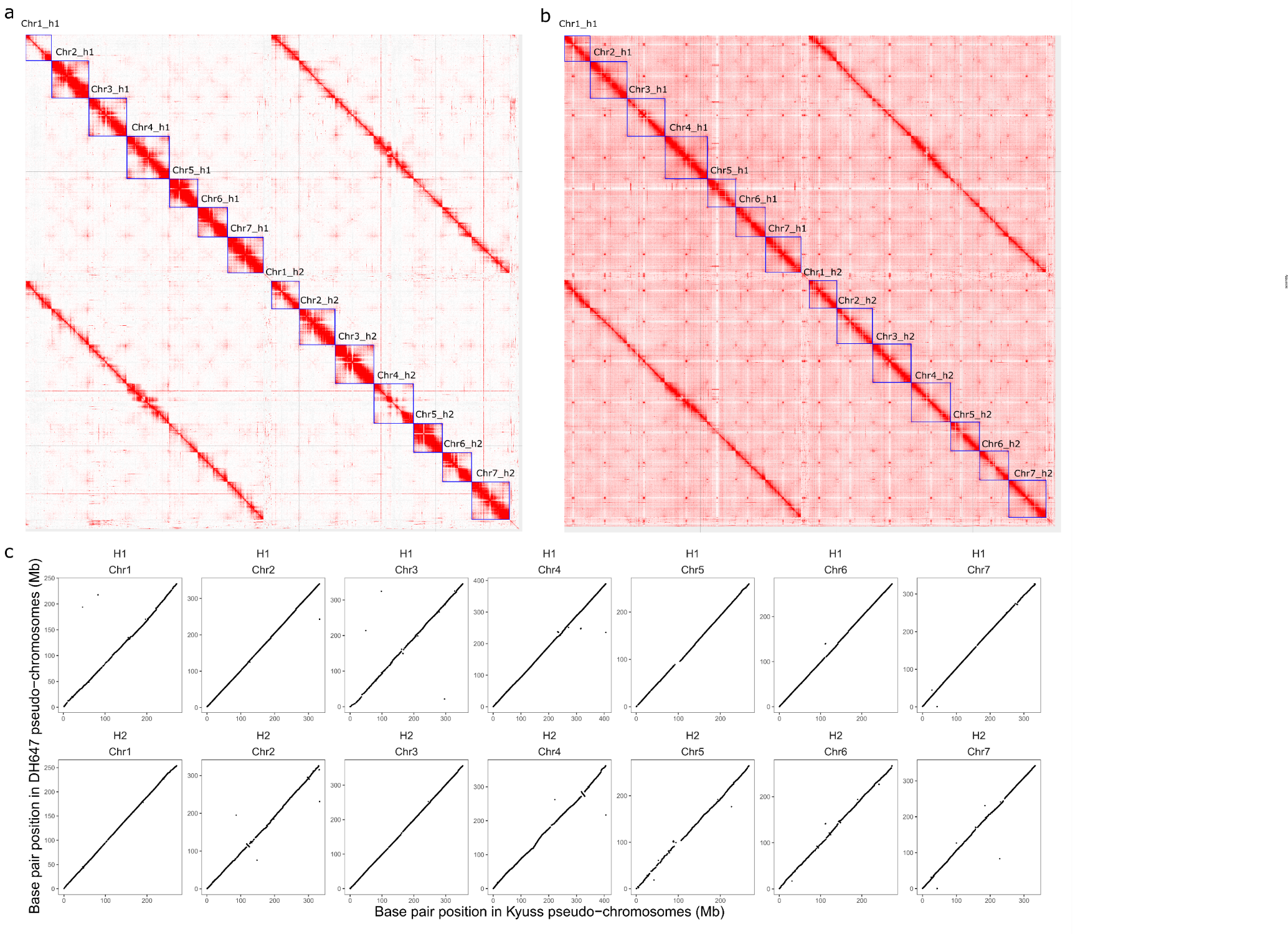


Supplementary Fig. 4. Haplotype-resolved pseudo-chromosomes (Chr) 1 to 7 of DH647. a, Hi-C contact map of the chromosome-level, haplotype-resolved assembly of DH647 with the Hi-C read mapping quality greater than 0. Less Hi-C signals were observed on Chr1_h1 and Chr4_h2. Hi-C reads could not be uniquely mapped between haplotypes of Chr1 or Chr4 due to the low heterozygosity. b, Hi-C contact map of the diploid assembly of DH647 with the Hi-C read mapping quality greater than or equal to 0. Without filtering the non-uniquely mapped Hi-C reads, more signals were observed in Chr1_h1 and Chr4_h2. c, Synteny between DH647 pseudo-chromosomes and Kyuss pseudo-chromosomes. Each dot represents an alignment of nonrepetitive sequence of DH647 against Kyuss. Only alignments equal to or longer than 2 kb with a mapping quality score of 60 were visualized. H1 and H2 represent haplome 1 and haplome 2 of DH647, respectively.


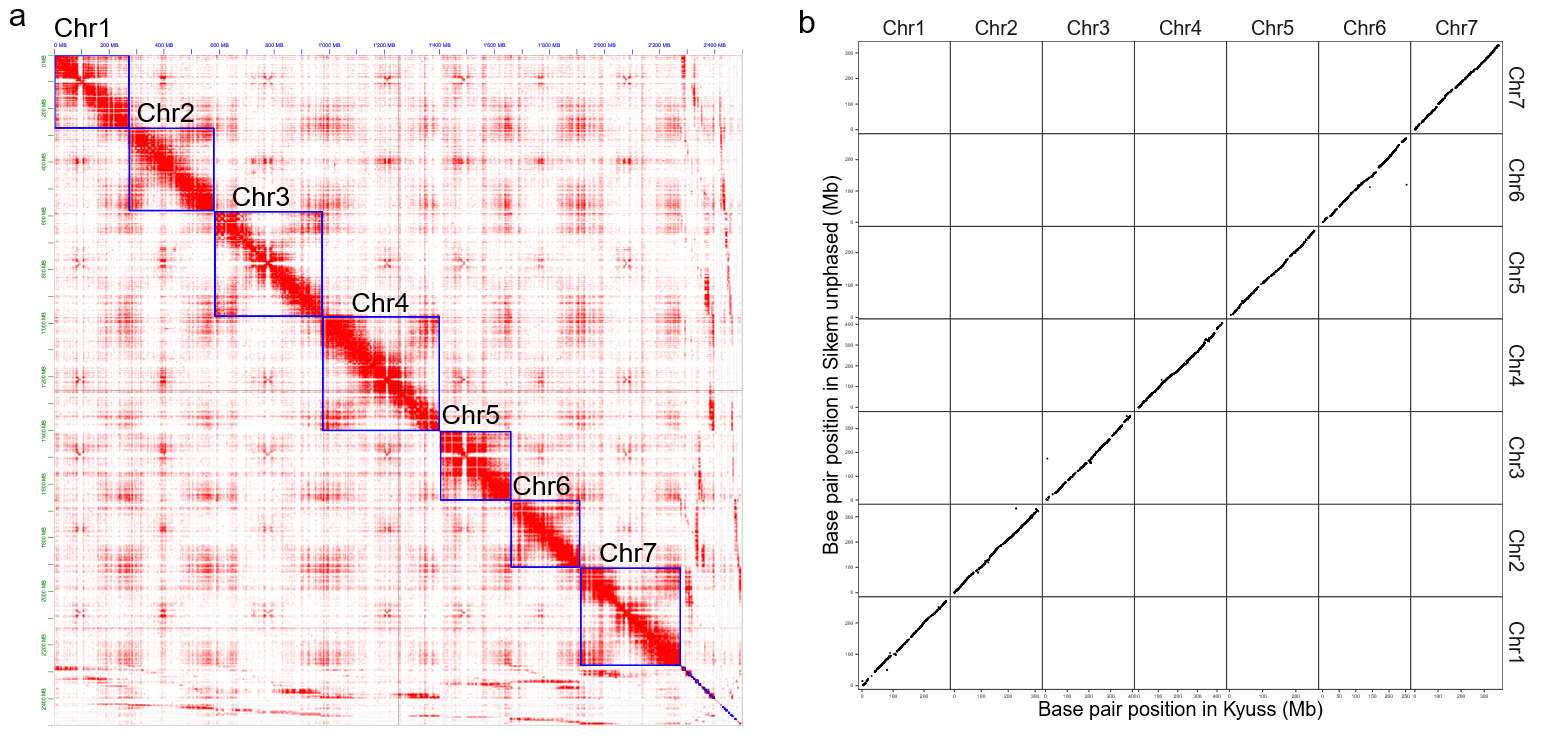


Supplementary Fig. 5. Pseudo-chromosomes of Sikem unphased haploid assembly. a, Hi-C contact map of the unphased haploid assembly of Sikem. b, Intra-chromosome synteny between Sikem haploid unphased assembly and Kyuss. Each dot represents an alignment, and only alignments equal to or longer than 2 kb with an alignment quality score of 60 were visualized.

Supplementary Fig. 6. Integrated Genome Browser (IGV) screenshot of Sikem phasing results of the two combinations of phasing data. a, Phasing with PacBio HiFi and Hi-C data. b, Phasing with ONT and Hi-C data. Panel a and b show the same 513 kb region on chromosome (Chr) 2 of the unphased haploid assembly of Sikem. PacBio HiFi reads resulted in small phase blocks (indicated by the red arrows in panel a) because: first, there were no Hi-C phased SNPs in the regions covered by the small phase blocks; second, PacBio HiFi reads were not long enough to connect SNPs in the small phase blocks with SNPs in the chromosome-level phase block (indicated by the black arrow in panel a). In contrast, ONT reads were long enough to bridge SNPs in the small phase blocks with SNPs in the chromosome-level phase block, producing only one phase block (indicated by the black arrow in panel b) in this region. Therefore, with the same density of Hi-C phased SNPs, longer reads produced a more contiguous phasing.


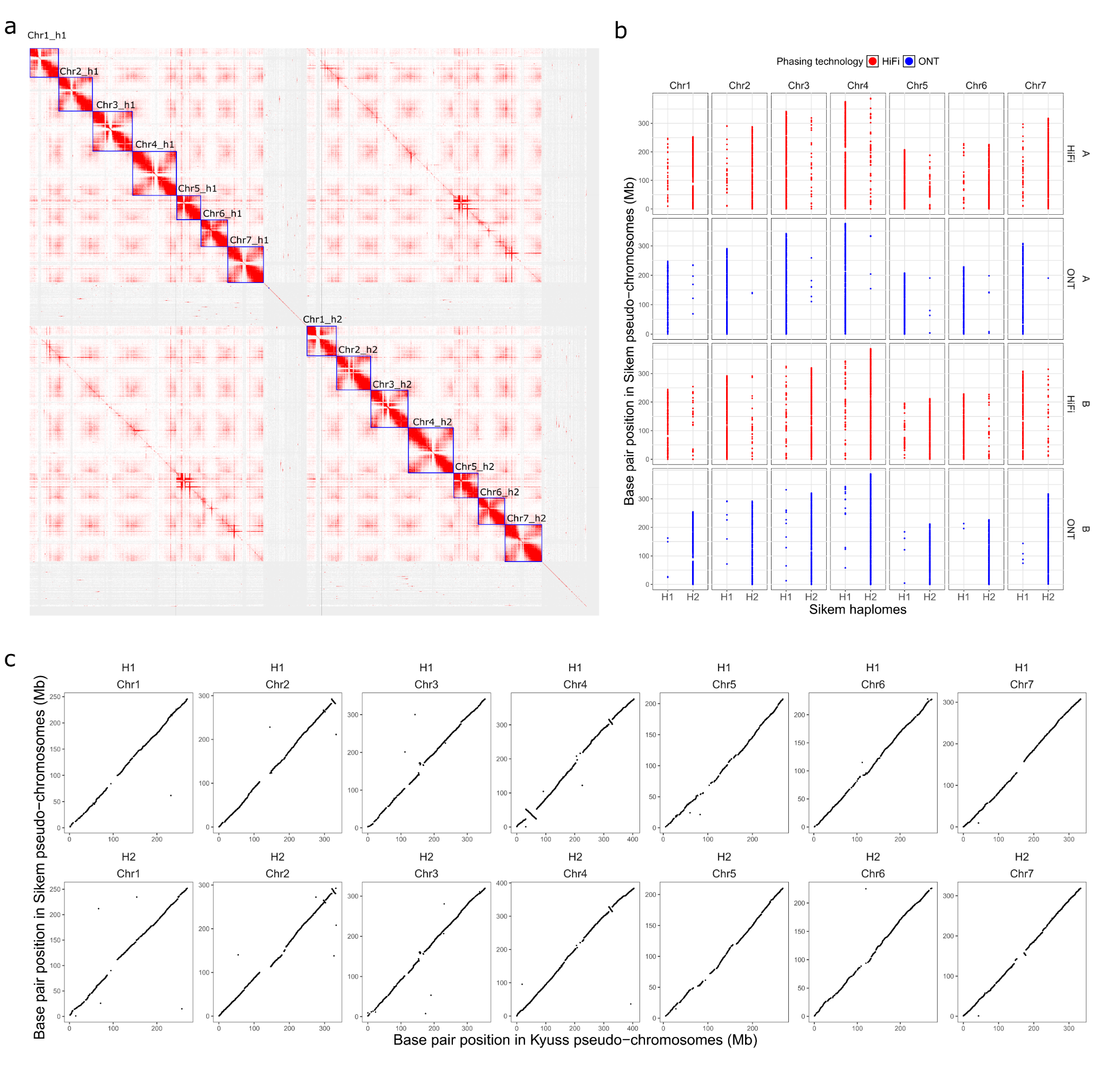


Supplementary Fig. 7. Sikem pseudo-chromosomes (Chr) 1 to 7. a, Hi-C contact map of the chromosome-level, haplotype-resolved assembly of Sikem (Hi-C read mapping quality score > 0). b, Mapping Sikem unitigs partitioned by different combinations of phasing data (HiFi-Hi-C in red and ONT-Hi-C in blue) to the final chromosome-level haplotype-resolved assembly of Sikem. Each dot in the plot represents one unitig. With either combination, unitigs were partitioned to A and B groups. When mapping either A or B group of unitigs from ONT-Hi-C to the assembly, most unitigs were mapped to the same haplotype for each homologous chromosome group. This was expected since the chromosome-level haplotype-resolved assembly was resulting from the unitigs partitioned by ONT-Hi-C. A few unitigs were mapped to the other haplotype. For these unitigs, the switch of haplotype was probably due to manual curation. When mapping either A or B groups of unitigs from HiFi-Hi-C to the assembly, much more unitigs were mapped to the other haplotype for each homologous chromosome group. The switch of haplotype for these unitigs might reflect the difference in phasing between the two combinations of phasing data. c, Synteny between Sikem pseudo-chromosomes and Kyuss pseudo-chromosomes. Each dot represents an alignment of nonrepetitive sequence of Sikem against Kyuss. Only alignments equal to or longer than 2 kb with a mapping quality score of or over 60 were visualized. H1 and H2 represent haplome 1 and haplome 2 of Sikem, respectively. Source data of plot b are provided as a Source Data file.


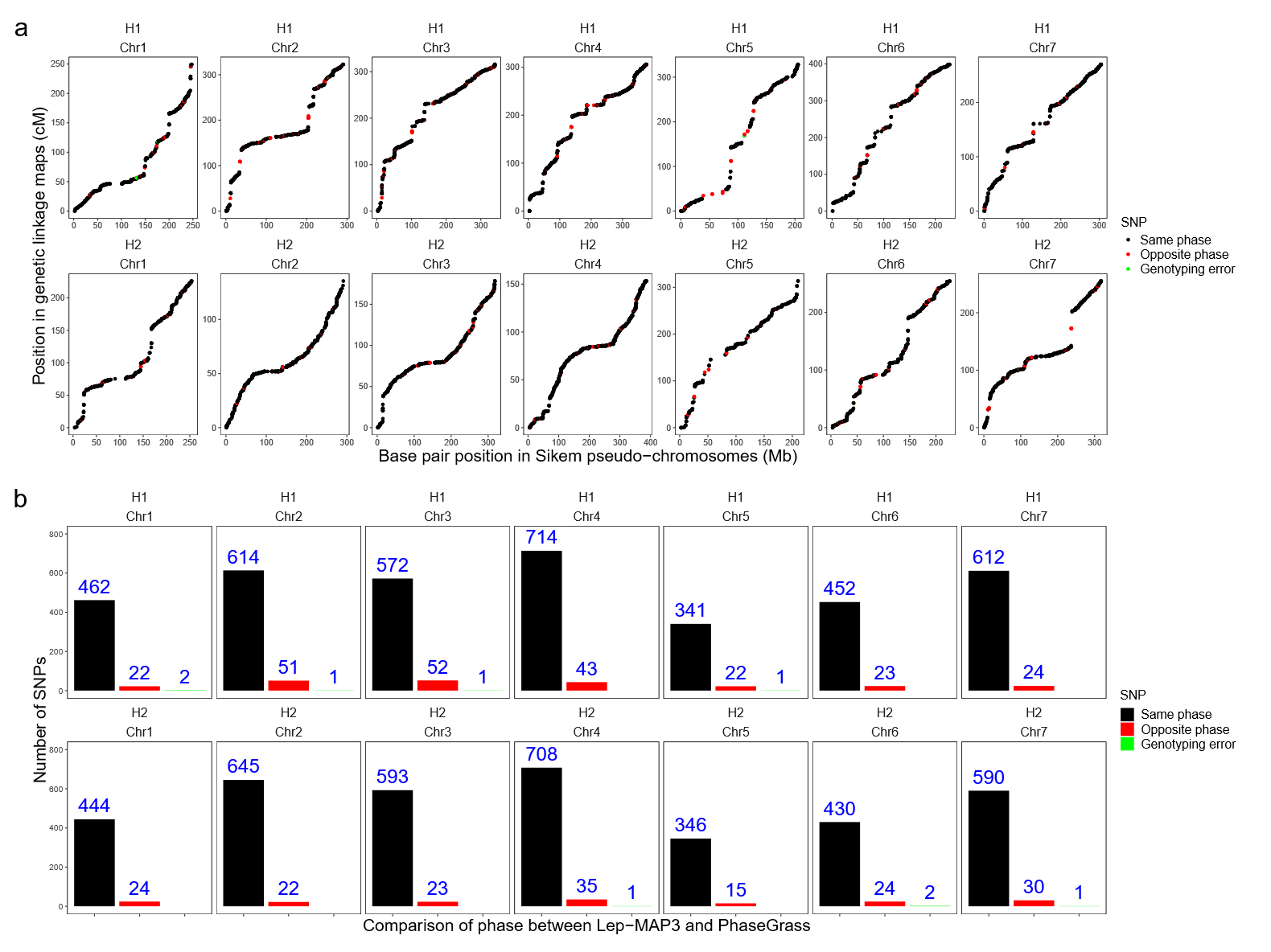


Supplementary Fig. 8. Validation of Sikem phasing using genetic linkage mapping. a, A genetic linkage map was constructed based on each haplome using Lep-MAP3. Each dot represents a SNP, and the position of the dot is determined by its base pair position on the pseudo-chromosome (Chr 1 to 7, x-axis) and its genetic linkage map position (y-axis). The color of the dot suggests the following: the black and the red color indicate that the haplotype from the genetic linkage map shared the same and the opposite allele with the haplome, respectively; The green color indicates that the allele in the genetic linkage map was different to both alleles in Sikem haplomes, suggesting a genotyping error. H1 and H2 represent haplome 1 and haplome 2 of Sikem, respectively. b, The number of the same, the opposite and incorrectly called alleles between the haplotype from PhaseGrass and the haplotype from the genetic linkage map. Source data are provided as a Source Data file. Source data are provided as a Source Data file.


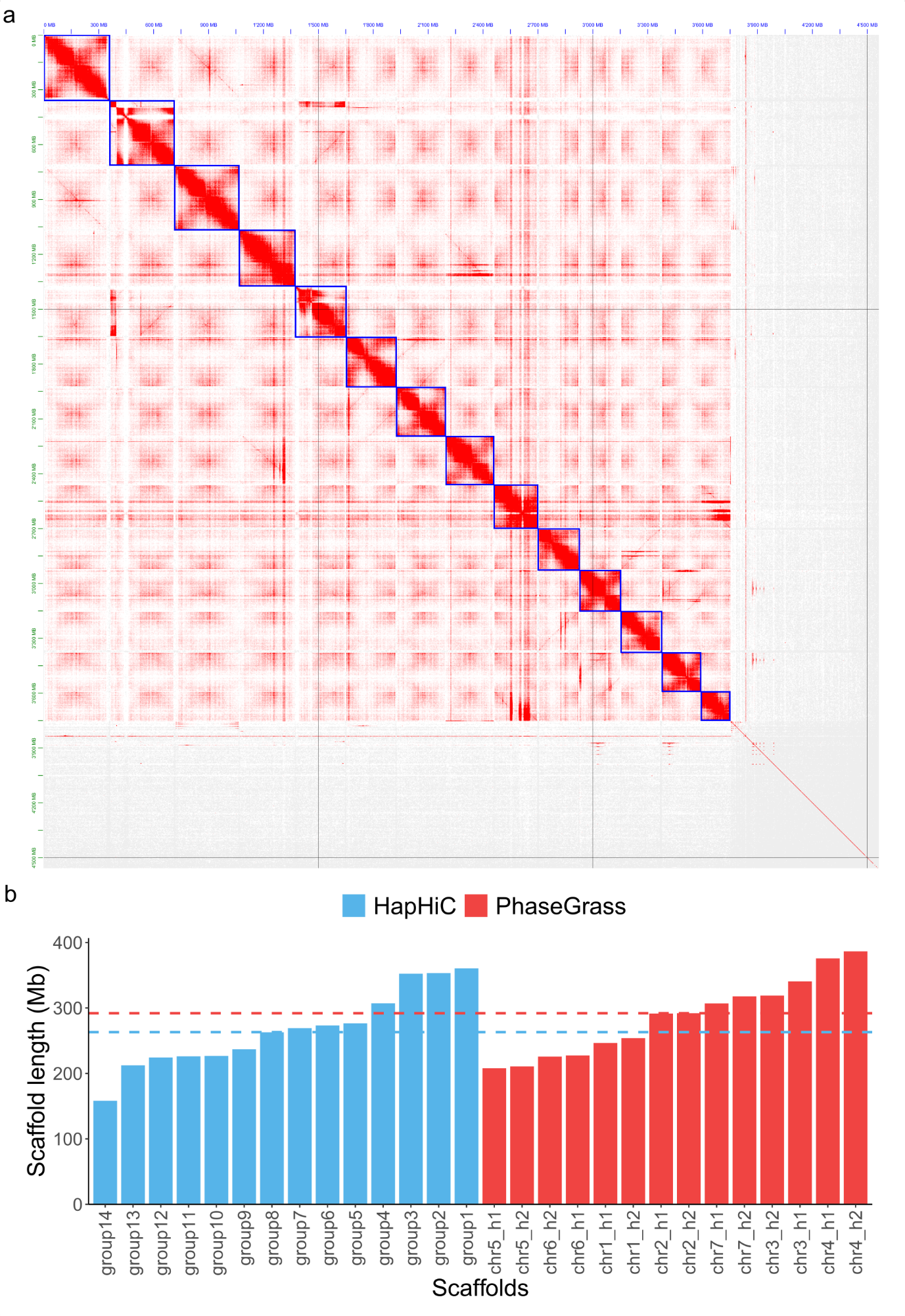


Supplementary Fig. 9. HapHiC results for Sikem based on the same unitig assembly and Hi-C data used for PhaseGrass. a, Hi-C contact map of scaffolds from Sikem generated by HapHiC (manual curation not applied). In total, 14 chromosome-level scaffolds (indicated as blue squares in the map) were obtained, corresponding to the 14 chromosomes in Sikem. However, homologous chromosome pairs were not identified, and the scaffolds were not partitioned to haplomes. Generally, the Hi-C contact map suggests very high scaffolding quality, and only a few scaffolding mistakes were observed. Further manual curation might improve the quality of the scaffolds. b, comparison of scaffold length between HapHiC and PhaseGrass. Only the longest 14 scaffolds were compared, and the dashed lines indicate the scaffold N50 of both methods (263 Mb from HapHiC vs 292 Mb from PhaseGrass). Overall, scaffolds from PhaseGrass (phased with ONT-Hi-C) were longer than scaffolds of HapHiC, and the total length of PhaseGrass pseudo-chromosomes was 262 Mb greater than that of HapHiC (4.02 Gb vs 3.75 Gb). Notably, the shortest HapHiC scaffold only spans 159 Mb. This may not be correct. Source data of plot b are provided as a Source Data file.


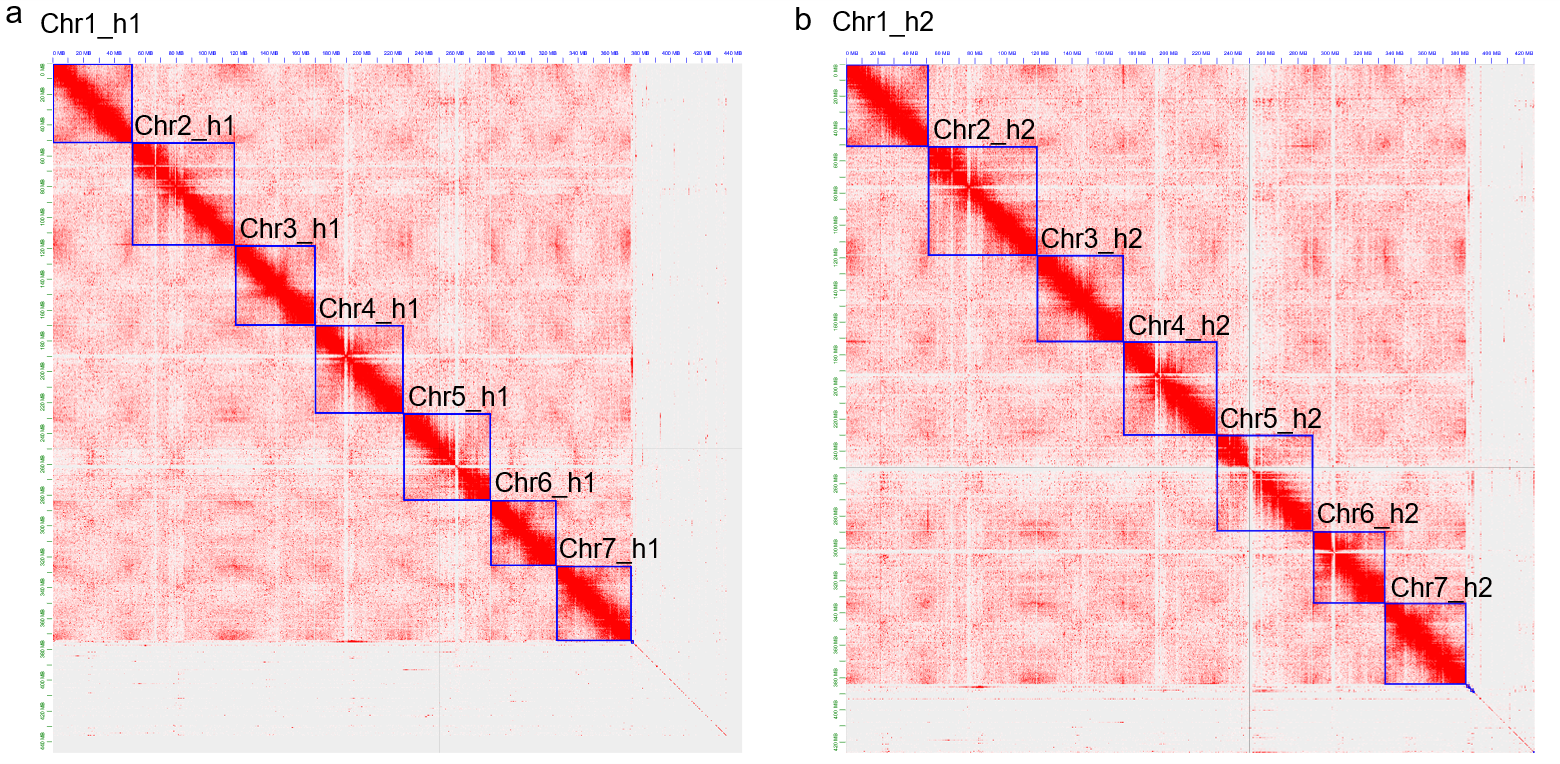


Supplementary Fig. 10. Hi-C contact map of HEN17 haplomes. a, Hi-C contact map of haplome 1. b, Hi-C contact map of haplome 2.


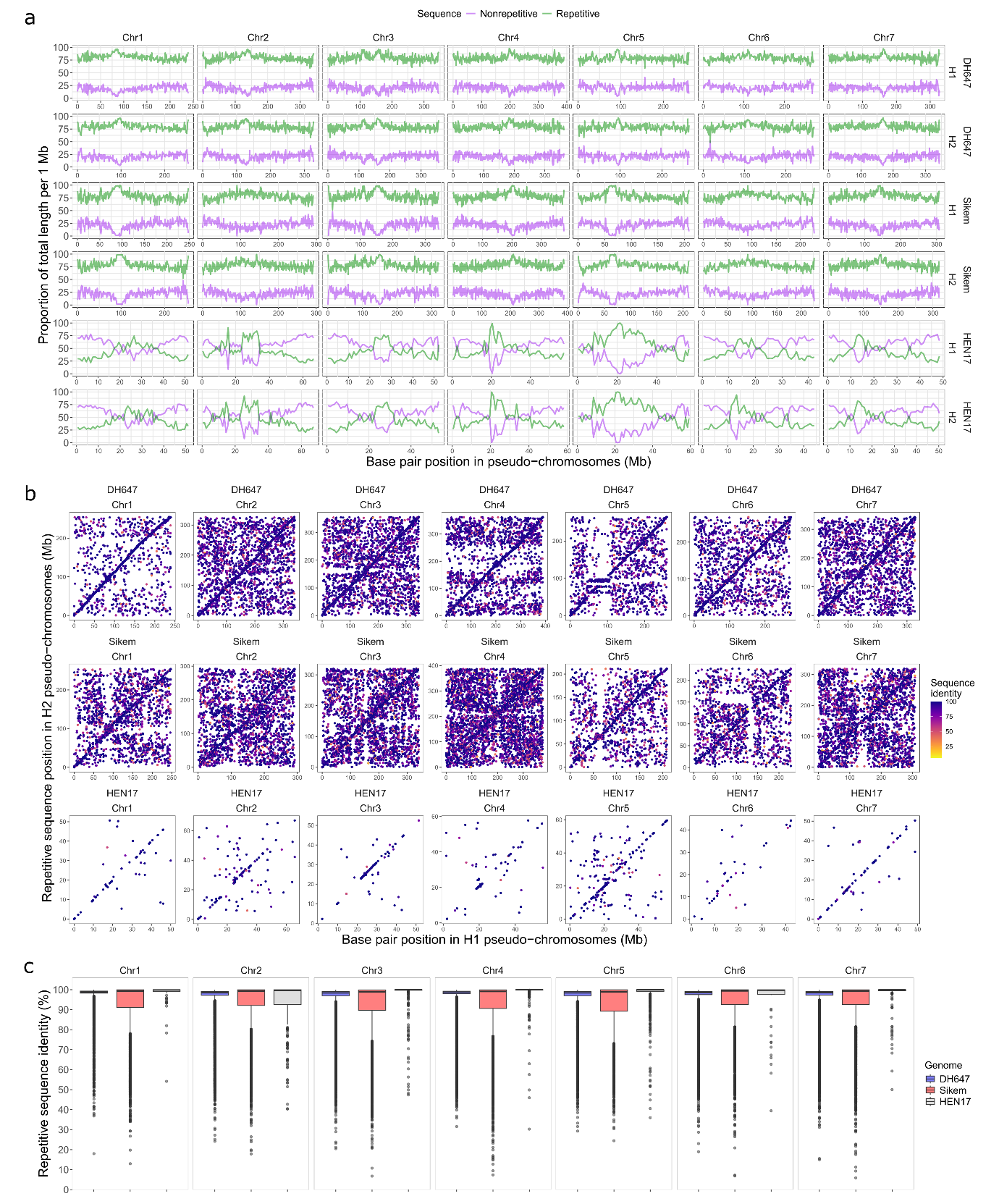


Supplementary Fig. 11. Nonrepetitive and repetitive sequences in DH647, Sikem and HEN17. a, Proportion of repetitive and nonrepetitive sequences in every 1 Mb of DH647, Sikem and HEN17 genomes. H1 and H2 represent two haplomes. b, Intra-chromosome dot plot of alignment of H2 repetitive sequences against H1. Each dot represents one alignment (mapping quality value > 0 and query length >= 2 kb). Each dot is colored according to their sequence identity. c, Distribution of sequence identity of repetitive sequences between H1 and H2 within chromosomes. Boxplots show median (black line), first and third quartile (hinges), 1.5 x inter-quartile-range (whiskers) and outliers (dots). Source data are provided as a Source Data file.

Supplementary Table 1. Sequencing data statistics.

| Sample | Sequencing data | Coverage per haplotype | Read length | Mean accuracy (QV) |
| --- | --- | --- | --- | --- |
| DH647 | WGS short reads | 26 × | 2 × 150 bp | 35 |
| DH647 | WGS ONT reads | 31 × | 30 kb (N50) | 11 |
| DH647 | Hi-C reads | 25 × | 2 × 110 bp | 35 |
| Sikem | WGS short reads | 26 × | 2 × 150 bp | 35 |
| Sikem | WGS ONT reads | 41 × | 46 kb (N50) | 10 |
| Sikem | WGS PacBio HiFi reads | 20 × | 16 kb (N50) | 33 |
| Sikem | Hi-C reads | 25 × | 2 × 110 bp | 35 |

| Sample | Chromosome (Chr) | Number of blocks | Heterozygous SNPs | Phased SNPs | Fraction of phased SNPs | Fraction of phased SNPs in the largest block | Phase block N50 (bp) |
| --- | --- | --- | --- | --- | --- | --- | --- |
| DH647 | Chr1 | 3 | 406’683 | 405’858 | 0.9980 | 0.9996 | 252’115’434 |
| DH647 | Chr2 | 2 | 1’114’329 | 1’112’094 | 0.9980 | 1.0000 | 328’061’788 |
| DH647 | Chr3 | 1 | 1’161’192 | 1’159’099 | 0.9982 | 1.0000 | 353’996’801 |
| DH647 | Chr4 | 1 | 786’817 | 785’377 | 0.9982 | 1.0000 | 406’061’665 |
| DH647 | Chr5 | 5 | 858’763 | 857’434 | 0.9985 | 1.0000 | 165’167’366 |
| DH647 | Chr6 | 1 | 864’878 | 862’985 | 0.9978 | 1.0000 | 274’008’430 |
| DH647 | Chr7 | 2 | 1’051’570 | 1’049’197 | 0.9977 | 1.0000 | 321’979’893 |
| DH647 | ALL | 15 | 6’244’232 | 6’232’044 | 0.9980 | 1.0000 | 321’979’893 |

Supplementary Table 2. DH647 phased SNPs with PhaseGrass.

| Sample | Chromosome (Chr) | Phase block | Start | End | SNPs (#) | Block length (bp) |
| --- | --- | --- | --- | --- | --- | --- |
| DH647 | Chr4 | Chromosome-level | 5’464 | 406’067’129 | 785’377 | 406’061’665 |
| DH647 | Chr3 | Chromosome-level | 28 | 353’996’829 | 1’159’099 | 353’996’801 |
| DH647 | Chr7 | Chromosome-level | 50 | 334’192’480 | 1’049’195 | 334’192’430 |
| DH647 | Chr2 | Chromosome-level | 12’341 | 333’278’978 | 1’112’078 | 333’266’637 |
| DH647 | Chr6 | Chromosome-level | 1’290 | 274’009’720 | 862’985 | 274’008’430 |
| DH647 | Chr1 | Chromosome-level | 421 | 271’784’613 | 405’705 | 271’784’192 |
| DH647 | Chr5 | Chromosome-level | 34’675 | 267’971’454 | 857’399 | 267’936’779 |
| DH647 | Chr2 | Small | 4’945’310 | 5’074’387 | 16 | 129’077 |
| DH647 | Chr1 | Small | 14’376’408 | 14’499’098 | 151 | 122’690 |
| DH647 | Chr5 | Small | 97’439’316 | 97’558’906 | 20 | 119’590 |
| DH647 | Chr7 | Small | 322’052’712 | 322’107’899 | 2 | 55’187 |
| DH647 | Chr5 | Small | 102’633’364 | 102’664’668 | 8 | 31’304 |
| DH647 | Chr5 | Small | 86’947’648 | 86’965’891 | 2 | 18’243 |
| DH647 | Chr1 | Small | 19’554’833 | 19’570’860 | 2 | 16’027 |
| DH647 | Chr5 | Small | 102’722’810 | 102’734’676 | 5 | 11’866 |

Supplementary Table 3. DH647 phase block information.

Supplementary Table 4. Sikem phased SNPs with PhaseGrass using different combinations of phasing data.

| Sample | Phasing data combination | Chromosome (Chr) | Number of blocks | Heterozygous SNPs | Phased SNPs | Fraction of phased SNPs | Fraction of phased SNPs in the largest block | Phase block N50 (bp) |
| --- | --- | --- | --- | --- | --- | --- | --- | --- |
| Sikem | PacBio HiFi + Hi-C | Chr1 | 301 | 810’188 | 809’804 | 0.9995 | 0.9968 | 2’077’164 |
| Sikem | PacBio HiFi + Hi-C | Chr2 | 294 | 1’040’644 | 1’040’277 | 0.9996 | 0.9978 | 2’506’660 |
| Sikem | PacBio HiFi + Hi-C | Chr3 | 469 | 1’113’894 | 1’113’292 | 0.9995 | 0.9960 | 1’784’116 |
| Sikem | PacBio HiFi + Hi-C | Chr4 | 433 | 1’267’363 | 1’266’626 | 0.9994 | 0.9974 | 1’861’339 |
| Sikem | PacBio HiFi + Hi-C | Chr5 | 377 | 663’288 | 663’006 | 0.9996 | 0.9944 | 1’426’537 |
| Sikem | PacBio HiFi + Hi-C | Chr6 | 274 | 816’528 | 816’204 | 0.9996 | 0.9974 | 1’736’030 |
| Sikem | PacBio HiFi + Hi-C | Chr7 | 422 | 1’028’587 | 1’028’063 | 0.9995 | 0.9966 | 1’749’442 |
| Sikem | PacBio HiFi + Hi-C | ALL | 2’570 | 6’740’492 | 6’737’272 | 0.9995 | 0.9967 | 1’851’222 |
| Sikem | ONT + Hi-C | Chr1 | 5 | 810’169 | 809’042 | 0.9986 | 1.0000 | 103’853’777 |
| Sikem | ONT + Hi-C | Chr2 | 3 | 1’040’635 | 1’039’444 | 0.9989 | 1.0000 | 192’822’645 |
| Sikem | ONT + Hi-C | Chr3 | 20 | 1’113’926 | 1’112’365 | 0.9986 | 0.9997 | 98’895’866 |
| Sikem | ONT + Hi-C | Chr4 | 2 | 1’267’361 | 1’265’529 | 0.9986 | 1.0000 | 297’711’388 |
| Sikem | ONT + Hi-C | Chr5 | 8 | 663’307 | 662’540 | 0.9988 | 0.9999 | 96’592’919 |
| Sikem | ONT + Hi-C | Chr6 | 4 | 816’583 | 815’616 | 0.9988 | 0.9999 | 85’147’584 |
| Sikem | ONT + Hi-C | Chr7 | 11 | 1’028’585 | 1’027’004 | 0.9985 | 0.9999 | 59’802’266 |
| Sikem | ONT + Hi-C | ALL | 53 | 6’740’566 | 6’731’540 | 0.9987 | 0.9999 | 103’853’777 |

Supplementary Table 5. HapHiC scaffolds.

| Scaffold | Length (bp) |
| --- | --- |
| group1 | 361506204 |
| group2 | 354276079 |
| group3 | 353276327 |
| group4 | 308104976 |
| group5 | 277545282 |
| group6 | 274311092 |
| group7 | 270118816 |
| group8 | 263816101 |
| group9 | 237925587 |
| group10 | 227762376 |
| group11 | 227133641 |
| group12 | 225251773 |
| group13 | 213334601 |
| group14 | 159116218 |

The remaining unanchored sequences not listed here are short unitigs, which can be seen from the Hi-C contact map, Supplementary Fig. 9a.

Supplementary Table 6. Assembly statistics of HEN17 chromosome-level haplotype-resolved assembly.

|  | Total length (bp) | Contig N50/N90 (Mb) | Contig L50/L90 (#) | Scaffold N50/N90 (Mb) | Scaffold L50/L90 (#) |
| --- | --- | --- | --- | --- | --- |
| Haplome 1 | 436’702’900 | 6.72/0.10 | 19/180 | 66.23/0.10 | 4/112 |
| Haplome 2 | 427’192’558 | 8.12/0.59 | 16/90 | 67.45/4.43 | 4/7 |

Supplementary Table 7. Proportion of nonrepetitive and repetitive sequences.

| Genome | Haplome | Sequence type | Fraction per 1Mb (%) |
| --- | --- | --- | --- |
| DH647 | H1 | Nonrepetitive | 20.50 |
| DH647 | H2 | Nonrepetitive | 20.50 |
| DH647 | H1 | Repetitive | 79.47 |
| DH647 | H2 | Repetitive | 79.47 |
| Sikem | H1 | Nonrepetitive | 21.42 |
| Sikem | H2 | Nonrepetitive | 21.66 |
| Sikem | H1 | Repetitive | 78.56 |
| Sikem | H2 | Repetitive | 78.32 |
| HEN17 | H1 | Nonrepetitive | 53.82 |
| HEN17 | H2 | Nonrepetitive | 52.92 |
| HEN17 | H1 | Repetitive | 46.18 |
| HEN17 | H2 | Repetitive | 47.08 |

Supplementary Table 8. Statistics of nonrepetitive and repetitive sequences (length >= 2 kb, mapping QV > 0).

| Genome | Sequence type | Number of sequences (#) | Total sequence length (bp) |
| --- | --- | --- | --- |
| DH647 | Nonrepetitive | 26’395 | 90’753’406 |
| DH647 | Repetitive | 67’759 | 254’814’063 |
| DH647 | Nonrepetitive not aligned | 5’713 | 19’292’265 |
| DH647 | Repetitive not aligned | - | - |
| Sikem | Nonrepetitive | 41’096 | 142’321’428 |
| Sikem | Repetitive | 169’823 | 902’845’780 |
| Sikem | Nonrepetitive not aligned | 9’328 | 32’568’465 |
| Sikem | Repetitive not aligned | 137 | 520’826 |
| HEN17 | Nonrepetitive | 17’154 | 55’865’165 |
| HEN17 | Repetitive | 7’248 | 51’618’794 |
| HEN17 | Nonrepetitive not aligned | 271 | 831’403 |
| HEN17 | Repetitive not aligned | 11 | 33’968 |

Supplementary Table 9. Type of variation in the alignment of nonrepetitive sequences.

| Genome | Chromosome (Chr) | SNV (#) | INDEL (#) | PAV (#) | SC (#) | SC >= 50 bp (#) |
| --- | --- | --- | --- | --- | --- | --- |
| DH647 | Chr1 | 70’424 | 19’926 | 257 | 1’308 | 806 |
| DH647 | Chr2 | 256’701 | 51’824 | 1’124 | 4’133 | 2’663 |
| DH647 | Chr3 | 254’594 | 49’805 | 1’094 | 4’044 | 2’632 |
| DH647 | Chr4 | 168’937 | 40’429 | 688 | 2’772 | 1’738 |
| DH647 | Chr5 | 206’856 | 39’004 | 909 | 3’267 | 2’132 |
| DH647 | Chr6 | 171’553 | 34’665 | 782 | 2’924 | 1’833 |
| DH647 | Chr7 | 216’088 | 44’017 | 968 | 3’579 | 2’333 |
| Sikem | Chr1 | 261’327 | 45’841 | 1’154 | 4’004 | 2’655 |
| Sikem | Chr2 | 359’447 | 63’089 | 1’525 | 5’245 | 3’514 |
| Sikem | Chr3 | 360’945 | 63’276 | 1’497 | 5’432 | 3’721 |
| Sikem | Chr4 | 418’932 | 74’964 | 1’680 | 6’383 | 4’228 |
| Sikem | Chr5 | 209’862 | 36’229 | 943 | 3’147 | 2’157 |
| Sikem | Chr6 | 250’058 | 44’376 | 1’067 | 3’766 | 2’527 |
| Sikem | Chr7 | 312’695 | 53’647 | 1’341 | 4’794 | 3’193 |
| HEN17 | Chr1 | 77’038 | 18’989 | 741 | 1’755 | 804 |
| HEN17 | Chr2 | 75’328 | 19’094 | 748 | 1’879 | 823 |
| HEN17 | Chr3 | 69’091 | 17’117 | 635 | 1’612 | 748 |
| HEN17 | Chr4 | 63’626 | 16’177 | 595 | 1’582 | 761 |
| HEN17 | Chr5 | 37’830 | 8’828 | 339 | 960 | 476 |
| HEN17 | Chr6 | 53’266 | 13’215 | 503 | 1’292 | 575 |
| HEN17 | Chr7 | 60’810 | 15’471 | 593 | 1’510 | 690 |
