# Supplementary material for: Chromosome-level haplotype-resolved assembly of highly heterozygous grass genomes with PhaseGrass
Source: Nat Commun. 2025 Dec 20;17:12. doi: 10.1038/s41467-025-66377-5 (PMC12764793; doi:10.1038/s41467-025-66377-5)
Supplement: Supplementary file 3 — Description of Additional Supplementary Files [file 41467_2025_66377_MOESM3_ESM.pdf]

Description of Additional Supplementary Files

File Name: Supplementary Data 1
Description: Sikem phase block information
