# Supplementary material for: Chromosome-level haplotype-resolved assembly of highly heterozygous grass genomes with PhaseGrass
Source: Nat Commun. 2025 Dec 20;17:12. doi: 10.1038/s41467-025-66377-5 (PMC12764793; doi:10.1038/s41467-025-66377-5)
Supplement: Supplementary file 5 — Reporting Summary [file 41467_2025_66377_MOESM5_ESM.pdf]

nature portfolio

Corresponding author(s): Bruno Studer, ETH Zurich, Switzerland
bruno.studer@usys.ethz.ch

Last updated by author(s): 31/10/25

Reporting Summary

Nature Portfolio wishes to improve the reproducibility of the work that we publish. This form provides structure for consistency and transparency in reporting. For further information on Nature Portfolio policies, see our Editorial Policies and the Editorial Policy Checklist.

Statistics

For all statistical analyses, confirm that the following items are present in the figure legend, table legend, main text, or Methods section.

n/a	Confirmed	
☐	☒	The exact sample size (*n*) for each experimental group/condition, given as a discrete number and unit of measurement
☒	☐	A statement on whether measurements were taken from distinct samples or whether the same sample was measured repeatedly
☒	☐	The statistical test(s) used AND whether they are one- or two-sided<br>*Only common tests should be described solely by name; describe more complex techniques in the Methods section.*
☒	☐	A description of all covariates tested
☒	☐	A description of any assumptions or corrections, such as tests of normality and adjustment for multiple comparisons
☐	☒	A full description of the statistical parameters including central tendency (e.g. means) or other basic estimates (e.g. regression coefficient) AND variation (e.g. standard deviation) or associated estimates of uncertainty (e.g. confidence intervals)
☒	☐	For null hypothesis testing, the test statistic (e.g. *F*, *t*, *r*) with confidence intervals, effect sizes, degrees of freedom and *P* value noted<br>*Give P values as exact values whenever suitable.*
☒	☐	For Bayesian analysis, information on the choice of priors and Markov chain Monte Carlo settings
☒	☐	For hierarchical and complex designs, identification of the appropriate level for tests and full reporting of outcomes
☒	☐	Estimates of effect sizes (e.g. Cohen's *d*, Pearson's *r*), indicating how they were calculated

Our web collection on statistics for biologists contains articles on many of the points above.

Software and code

Policy information about availability of computer code

Data collection	No software was used
Data analysis	Hifiasm v0.19.8, NextDenovo v2.5.2, NextPolish v1.4.1, BUSCO v5.3.2, GMAP 2021.08.25, MCScanX v1.0.0, TRITEX, Juicer v2.0, 3D-DNA, JuiceBox, v1.11.08, Snakemake v7.20.0, BWA v0.7.17, SAMTools v1.17, BCFTools v1.17, freebayes v1.3.6, HapCut2 v1.3.3, Winnowmap2 v2.03, WhatsHap v1.7, KMC v3.2.1, trio_binning v1.0.0, seqkit v2.4.0, Hapo-G v1.3.7, YaHS v1.2a.2, RagTag v2.1.0, R v4.5.0, LepMAP3 v0.5, KAT v2.4.1, BEDTools v2.30.0, POLCA v4.1.0, mosdepth v0.3.7, minimap2 v2.26, MUMmer v4.00rcl, assembly-stats v1.0.0

For manuscripts utilizing custom algorithms or software that are central to the research but not yet described in published literature, software must be made available to editors and reviewers. We strongly encourage code deposition in a community repository (e.g. GitHub). See the Nature Portfolio guidelines for submitting code & software for further information.

Data

Policy information about availability of data

All manuscripts must include a data availability statement. This statement should provide the following information, where applicable:

- Accession codes, unique identifiers, or web links for publicly available datasets
- A description of any restrictions on data availability
- For clinical datasets or third party data, please ensure that the statement adheres to our policy

The chromosome-level, haplotype-resolved assemblies of DH647 and Sikem are available on NCBI under BioProject PRJNA1147195 (DH647 h1), PRJNA1147194 (DH647 h2), PRJNA1147193 (Sikem h1) and PRJNA1147192 (Sikem h2). All genome sequencing data generated in this study, including ONT reads of DH647 and

April 2023

Sikem, PacBio HiFi reads of Sikem, Hi-C reads and WGS short reads of DH647 and Sikem, are available on NCBI under BioProject PRJNA1156640 and PRJNA1156633, respectively. The red clover HEN17 haplotype-resolved assembly generated in this study is available at Zenodo, 10.5281/zenodo.15411222.

Research involving human participants, their data, or biological material

Policy information about studies with human participants or human data. See also policy information about sex, gender (identity/presentation), and sexual orientation and race, ethnicity and racism.

Reporting on sex and gender	*Use the terms sex (biological attribute) and gender (shaped by social and cultural circumstances) carefully in order to avoid confusing both terms. Indicate if findings apply to only one sex or gender; describe whether sex and gender were considered in study design; whether sex and/or gender was determined based on self-reporting or assigned and methods used. Provide in the source data disaggregated sex and gender data, where this information has been collected, and if consent has been obtained for sharing of individual-level data; provide overall numbers in this Reporting Summary. Please state if this information has not been collected. Report sex- and gender-based analyses where performed, justify reasons for lack of sex- and gender-based analysis.*
Reporting on race, ethnicity, or other socially relevant groupings	*Please specify the socially constructed or socially relevant categorization variable(s) used in your manuscript and explain why they were used. Please note that such variables should not be used as proxies for other socially constructed/relevant variables (for example, race or ethnicity should not be used as a proxy for socioeconomic status). Provide clear definitions of the relevant terms used, how they were provided (by the participants/respondents, the researchers, or third parties), and the method(s) used to classify people into the different categories (e.g. self-report, census or administrative data, social media data, etc.) Please provide details about how you controlled for confounding variables in your analyses.*
Population characteristics	*Describe the covariate-relevant population characteristics of the human research participants (e.g. age, genotypic information, past and current diagnosis and treatment categories). If you filled out the behavioural & social sciences study design questions and have nothing to add here, write "See above."*
Recruitment	*Describe how participants were recruited. Outline any potential self-selection bias or other biases that may be present and how these are likely to impact results.*
Ethics oversight	*Identify the organization(s) that approved the study protocol.*

Note that full information on the approval of the study protocol must also be provided in the manuscript.

Field-specific reporting

Please select the one below that is the best fit for your research. If you are not sure, read the appropriate sections before making your selection.

☐ Life sciences ☐ Behavioural & social sciences ☒ Ecological, evolutionary & environmental sciences

For a reference copy of the document with all sections, see nature.com/documents/nr-reporting-summary-flat.pdf

Ecological, evolutionary & environmental sciences study design

All studies must disclose on these points even when the disclosure is negative.

Study description	Whole-genome sequencing of highly heterozygous Lolium multiflorum and L. perenne genotypes, along with the development of an assembly, phasing, and scaffolding workflow tailored for these species or others with similarly high levels of heterozygosity.
Research sample	DH647 is a L. perenne genotype derived from a doubled haploid induction program. Sikem is a genotype from the L. multiflorum cultivar 'Sikem' developed by DLF A/S, Denmark.
Sampling strategy	A clone of DH647 was selected for sequencing. A genotype (M.2002/18) of the cultivar 'Sikem' was selected for sequencing.
Data collection	ONT long reads for both DH647 and Sikem were obtained at Agroscope, Switzerland, using a published in-house sequencing protocol. PacBio HiFi data of Sikem were generated by Arizona Genomics Institute, Tucson, AZ. Hi-C data for both samples were generated by IPK, Gatersleben, Germany. Illumina WGS pair-end short reads for both samples were generated by Functional Genomics Center Zurich, Switzerland.
Timing and spatial scale	Sequencing data collection for this study was carried out between 2019 and 2022. The timeline was determined by the availability of plant materials, the readiness and optimization of laboratory protocols, and sequencing capacities at the collaborating institutions.
Data exclusions	No data were excluded from the analysis.
Reproducibility	The outcome of this work can be successfully reproduced by applying the raw data to the described workflow, as both are publicly available.
Randomization	*Describe how samples/organisms/participants were allocated into groups. If allocation was not random, describe how covariates were controlled. If this is not relevant to your study, explain why.*
Blinding	*Describe the extent of blinding used during data acquisition and analysis. If blinding was not possible, describe why OR explain why*

blinding was not relevant to your study.

Did the study involve field work? ☐ Yes ☒ No

Reporting for specific materials, systems and methods

We require information from authors about some types of materials, experimental systems and methods used in many studies. Here, indicate whether each material, system or method listed is relevant to your study. If you are not sure if a list item applies to your research, read the appropriate section before selecting a response.

Materials & experimental systems

n/a	Involved in the study	
☒	☐	Antibodies
☒	☐	Eukaryotic cell lines
☒	☐	Palaeontology and archaeology
☒	☐	Animals and other organisms
☒	☐	Clinical data
☒	☐	Dual use research of concern
☐	☒	Plants

Methods

n/a	Involved in the study	
☒	☐	ChIP-seq
☒	☐	Flow cytometry
☒	☐	MRI-based neuroimaging

Dual use research of concern

Policy information about dual use research of concern

Hazards

Could the accidental, deliberate or reckless misuse of agents or technologies generated in the work, or the application of information presented in the manuscript, pose a threat to:

No	Yes	
☒	☐	Public health
☒	☐	National security
☒	☐	Crops and/or livestock
☒	☐	Ecosystems
☒	☐	Any other significant area

Experiments of concern

Does the work involve any of these experiments of concern:

No	Yes	
☒	☐	Demonstrate how to render a vaccine ineffective
☒	☐	Confer resistance to therapeutically useful antibiotics or antiviral agents
☒	☐	Enhance the virulence of a pathogen or render a nonpathogen virulent
☒	☐	Increase transmissibility of a pathogen
☒	☐	Alter the host range of a pathogen
☒	☐	Enable evasion of diagnostic/detection modalities
☒	☐	Enable the weaponization of a biological agent or toxin
☒	☐	Any other potentially harmful combination of experiments and agents

Plants

Seed stocks	The sequenced genotypes are maintained by the Molecular Plant Breeding group of ETH Zurich, at the ETH Research Station for Plant Sciences in Eschikon, Zurich, Switzerland.
Novel plant genotypes	*Describe the methods by which all novel plant genotypes were produced. This includes those generated by transgenic approaches, gene editing, chemical/radiation-based mutagenesis and hybridization. For transgenic lines, describe the transformation method, the number of independent lines analyzed and the generation upon which experiments were performed. For gene-edited lines, describe the editor used, the endogenous sequence targeted for editing, the targeting guide RNA sequence (if applicable) and how the editor was applied.*
Authentication	*Describe any authentication procedures for each seed stock used or novel genotype generated. Describe any experiments used to assess the effect of a mutation and, where applicable, how potential secondary effects (e.g. second site T-DNA insertions, mosiacism, off-target gene editing) were examined.*
